# Field emission properties and growth mechanism of In_2_O_3_ nanostructures

**DOI:** 10.1186/1556-276X-9-111

**Published:** 2014-03-10

**Authors:** Bing Wang, Zhaoqiang Zheng, Huanyu Wu, Lianfeng Zhu

**Affiliations:** 1 Shenzhen Key Lab of Micro-Nano Photonic Information Technology, College of Electronic Science and Technology, Shenzhen University, Nanhai Ave 3688, Shenzhen, Guangdong 518060, People’s Republic of China; 2 Department of Materials Science and Engineering, Tsinghua University, Beijing 100084, People’s Republic of China

**Keywords:** Thermal evaporation, Field emission, Crystal growth, Growth mechanism

## Abstract

Four kinds of nanostructures, nanoneedles, nanohooks, nanorods, and nanotowers of In_2_O_3_, have been grown by the vapor transport process with Au catalysts or without any catalysts. The morphology and structure of the prepared nanostructures are determined on the basis of field emission scanning electron microscopy (FESEM), x-ray diffraction (XRD), and transmission electron microscopy (TEM). The growth direction of the In_2_O_3_ nanoneedles is along the [001], and those of the other three nanostructures are along the [100]. The growth mechanism of the nanoneedles is the vapor-liquid–solid (VLS), and those of the other three nanostructures are the vapor-solid (VS) processes. The field emission properties of four kinds of In_2_O_3_ nanostructures have been investigated. Among them, the nanoneedles have the best field emission properties with the lowest turn-on field of 4.9 V/μm and the threshold field of 12 V/μm due to possessing the smallest emitter tip radius and the weakest screening effect.

## Background

Indium oxide (In_2_O_3_) is a wide-band-gap semiconducting oxide that has been used for transparent conducting oxides because of its high conductivity and transparency [1-3].

Recent reports show that reducing the size of In_2_O_3_ to a nanoscale gives it various morphologies, such as wires/belts, cubes, octahedrons, and bamboos [3-7]. Recently, the nanostructures of In_2_O_3_ have also been paid considerable attention due to their esthetic morphologies [6], novel characteristics, and important potential applications in various nanodevices [8-13]. It is well known that the properties of nanostructures strongly depend on their morphologies. In previous reports, most of the efforts were focused on the synthesis and properties of single morphology nanostructures. Research on the complex nanostructure was limited, while investigation of the synthesis and properties of complex nanostructures represented developing directions of nanoscience and nanotechnology, which have important potential applications in realizing the multiple functions of nanodevices [14].

Field emission is one of the most fascinating properties of nanomaterials, such as carbon nanotube, ZnO nanoneedles, and SnO_2_ nanograss [15-19], and has been extensively studied due to its diverse technological applications in flat-panel displays, microwave-generation devices, and vacuum micro/nanoelectronic devices [20]. In_2_O_3_ can be one of the most attractive conductive oxides for field emission because of its relatively low electron affinity, convenience of n-type doping, high chemical inertness, and sputter resistance [21].

In this paper, four kinds of In_2_O_3_ structures, nanoneedles, nanohooks, nanorods, and nanotowers have been grown by the vapor transport process. The morphology and structure of the prepared nanostructures are determined on the basis of field emission scanning electron microscopy (FESEM), x-ray diffraction (XRD), and transmission electron microscopy (TEM). The field emission properties of the four kinds of In_2_O_3_ nanostructures have been investigated, and the In_2_O_3_ nanoneedles have preferable characteristics among the four nanostructures due to possessing the smallest emitter tip radius and the weakest screening effect. The growth mechanism is discussed, and the analysis is helpful to understand the relationship between the kinetic factors and the complex structures. It is valuable to realize the controlled synthesis of complex nanostructures.

## Methods

The synthesis of these In_2_O_3_ nanostructures is by the vapor transport process. The fabrication of the In_2_O_3_ nanoneedles is as follows: the Au layer (about 10 nm in thickness) is deposited on one single crystal silicon (001) substrate with area of 5 mm^2^ by sputtering. The active carbon and In_2_O_3_ powders (both 99.99%) are mixed in a 1:1 weight ratio and placed into a small quartz tube. One Si substrate covered by Au is put near the mixture of carbon and In_2_O_3_ inside the small quartz tube. Then the small quartz tube is pulled into a large quartz tube, and the large quartz tube is put in an electric furnace. The whole system is evacuated by a vacuum pump for 20 min, then the argon gas is guided into the system at 200 sccm, and the pressure is kept at 300 Torr. Afterwards, the system is rapidly heated up to 1,000°C from the room temperature and kept at the temperature for 1 h. Finally, the system is cooled down to the room temperature in several hours. When the substrate is taken out, we can see yellow products on the substrate.

The fabrication process of the In_2_O_3_ nanohooks, In_2_O_3_ nanorods, and In_2_O_3_ nanotowers is basically same with that of In_2_O_3_ nanoneedles besides the following contents: Three Si substrates without any catalysts are put far away from the mixture of carbon and In_2_O_3_ inside the small quartz tube, and the distance between every two Si substrates is about 2 cm. The argon gas is guided into the system at 250 sccm, the pressure is kept at 350 Torr, and the system is rapidly heated up to 1,050°C from the room temperature.

FESEM, XRD, and TEM are employed to identify the morphology and structure of the synthesized productions. Note that we can easily repeat the experimental results, suggesting that our method is flexible and reproducible.

## Results and discussion

**Figure 1:**
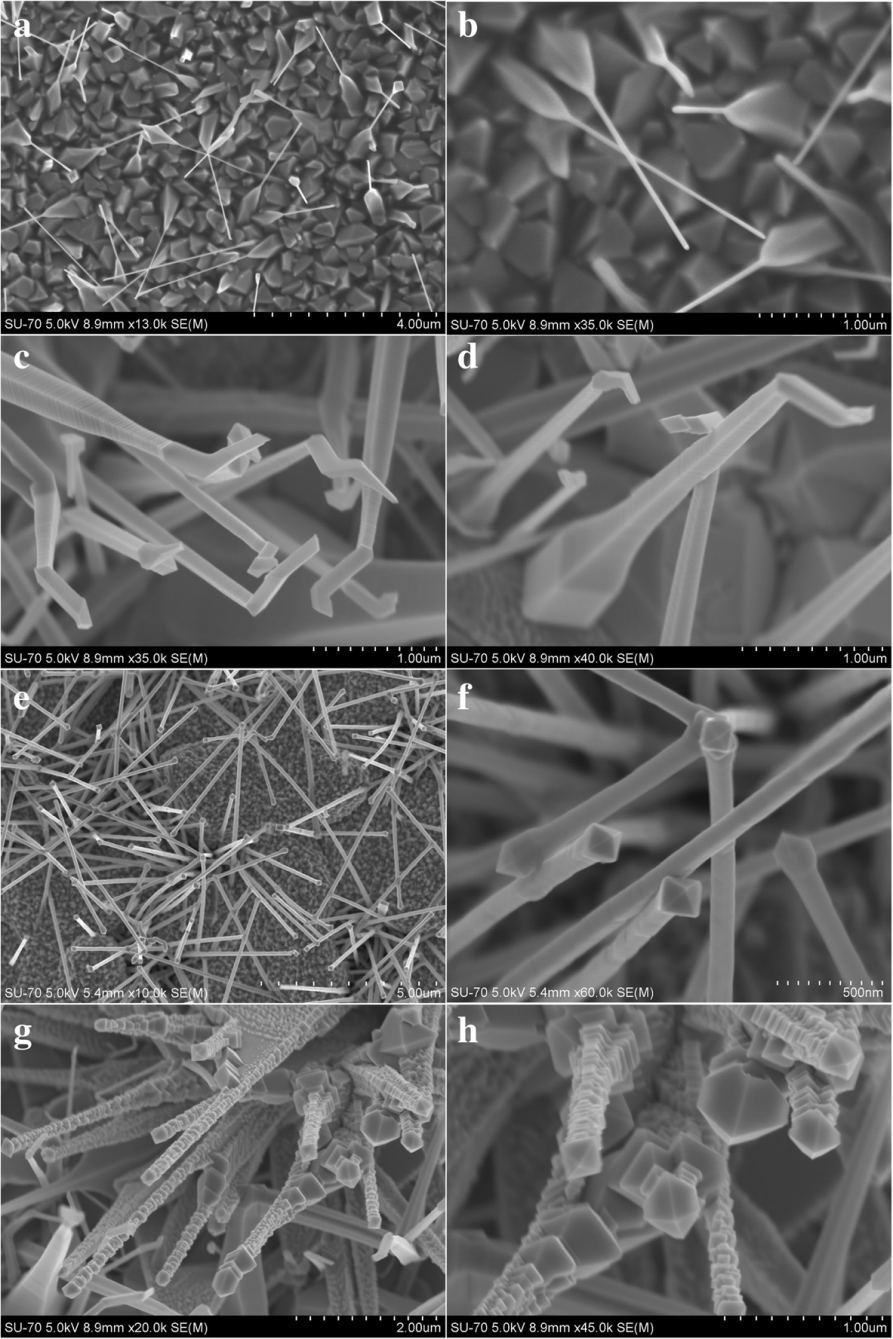
**Morphologies of the synthesized In**_**2**_**O**_**3 **_**nanostructures. (a,b)** Low- and high-magnified FESEM image of In_2_O_3_ nanoneedles. **(c,d)** High-magnified FESEM images of In_2_O_3_ nanohooks. **(e,f)** Low- and high-magnified FESEM image of In_2_O_3_ nanorods. **(g,h)** High-magnified FESEM images of In_2_O_3_ nanotowers.

The morphologies of the synthesized In_2_O_3_ nanostructures are shown as Figure 1. The low-magnified FESEM image of the In_2_O_3_ nanoneedles is shown in Figure 1a. The as-synthesized In_2_O_3_ nanoneedles consist of a short thick section and a long thin section. The high-magnified FESEM image in Figure 1b shows that several In_2_O_3_ nanoneedles consist of a short thick and pencil-like section with an average diameter of 150 to 200 nm, and a long thin and needle-like section with an average diameter of 50 nm. Figure 1c,d shows the high-magnified FESEM images of the In_2_O_3_ nanohooks. The nanohooks consist of a layer-shaped section with the size of 200 nm and a hook-like section with the tip size of 100 nm. Figure 1e shows the low-magnified FESEM image of the In_2_O_3_ nanorods. The high-magnified FESEM image in Figure 1f shows that several In_2_O_3_ nanorods consist of a layer-shaped section with the size of 100 nm and an imperfect octahedral cap with the size of 125 nm. Figure 1g,h shows the high-magnified FESEM images of the In_2_O_3_ nanotowers. The four sides of the nanotower are chucked up with octahedrons one after another so that the nanotower is with a decreasing size from the bottom to the top. The top of the nanotower is an octahedral cap with the size of 300 to 600 nm, and the size of 300 nm is dominant. The length of the four kinds of In_2_O_3_ nanostructures in Figure 1 is all close to 2 μm.

**Figure 2:**
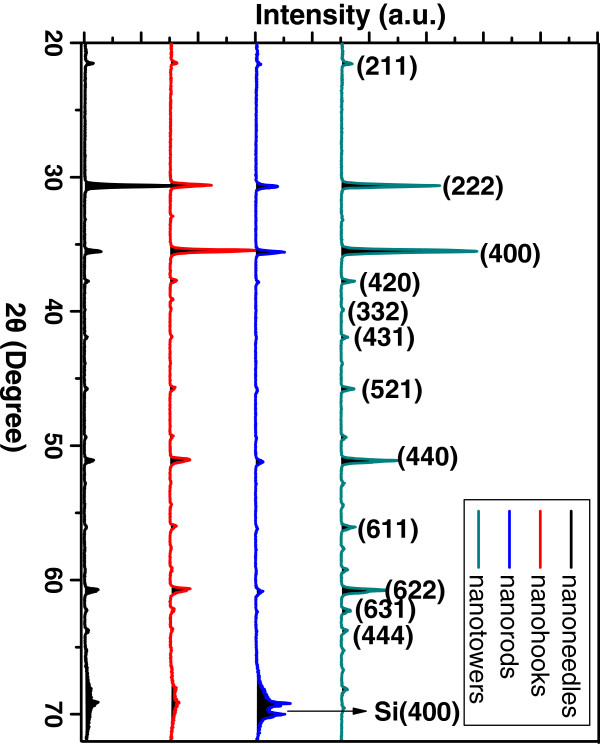
**The XRD pattern of four kinds of In**_**2**_**O**_**3 **_**nanostructures.**

The corresponding XRD pattern of the samples in Figure 2 shows that the fabricated nanostructures are indexed to the cubic In_2_O_3_. According to PDF no. 06-0416, the lattice constant of the cubic In_2_O_3_ are *a* = 10.118 Å, *b* = 10.118 Å, and *c* = 10.118 Å, respectively.

**Figure 3:**
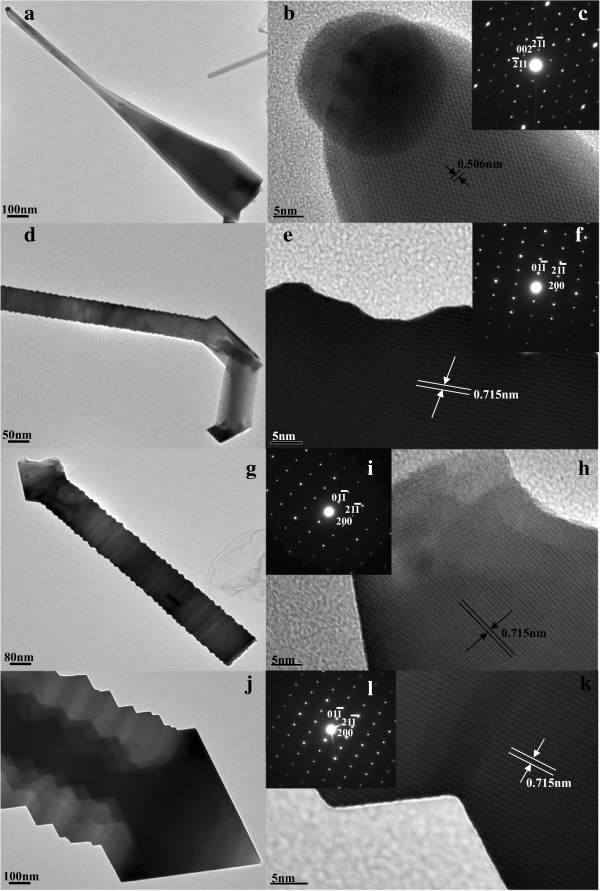
**Analysis and morphology and structure of the as-synthesized samples. (a,b,c)** TEM bright-field image, HRTEM image, and corresponding SAED pattern of individual In_2_O_3_ nanoneedles. **(d,e,f)** TEM bright-field image, the HRTEM image, and the corresponding SAED pattern of individual In_2_O_3_ nanohooks. **(g,h,i)** TEM bright-field image, the HRTEM image, and the corresponding SAED pattern of individual In_2_O_3_ nanorods. **(j,k,l)** TEM bright-field image, the HRTEM image, and the corresponding SAED pattern of individual In_2_O_3_ nanotowers.

The morphology and structure of the as-synthesized samples are analyzed in detail by TEM in Figure 3. The typical TEM bright-field image of an individual In_2_O_3_ nanoneedle with tip width of 50 nm is shown in Figure 3a. The high-resolution transmission electron microscopy (HRTEM) image shown in Figure 3b is recorded at the tip of the In_2_O_3_ nanoneedle in Figure 3a. The interplanar spacing of 0.506 nm is corresponding to the (002) crystallographic plane of cubic In_2_O_3_ lattice. In addition, the black ball in the tip of the In_2_O_3_ nanoneedle is the Au catalyst. The corresponding selected area electronic diffraction (SAED) pattern in Figure 3c recorded with an electron beam perpendicular to the surface of the In_2_O_3_ nanoneedle demonstrates that the In_2_O_3_ nanoneedle is a single crystal and the growth direction is along [002]. Figure 3d is a typical TEM bright-field image of an individual In_2_O_3_ nanohook with tip width of 100 nm. The HRTEM image shown in Figure 3e is recorded at the boundary of the layer-shaped section in the In_2_O_3_ nanohook in Figure 3d. The interplanar spacing of 0.715 nm is corresponding to the (011) crystallographic plane of cubic In_2_O_3_ lattice, and the corresponding SAED pattern in Figure 3f recorded with an electron beam perpendicular to the surface of the In_2_O_3_ nanohook demonstrates that the In_2_O_3_ nanohook is a single crystal and the growth direction is along [200]. Figure 3g is a typical TEM bright-field image of an individual In_2_O_3_ nanorod with octahedral cap size of 125 nm. The HRTEM image shown in Figure 3h is recorded at the octahedral cap of the In_2_O_3_ nanorod in Figure 3g. The interplanar spacing of 0.715 nm is corresponding to the (011) crystallographic plane of cubic In_2_O_3_ lattice, and the corresponding SAED pattern in Figure 3i recorded with an electron beam perpendicular to the surface of the In_2_O_3_ nanorod demonstrates that the In_2_O_3_ nanorod is a single crystal and the growth direction is along [200]. Figure 3j is a typical TEM bright-field image of an individual In_2_O_3_ nanotower with octahedral cap size of 600 nm. The HRTEM image shown in Figure 3k is recorded at the body section of the In_2_O_3_ nanotower in Figure 3j. The interplanar spacing of 0.715 nm is corresponding to the (011) crystallographic plane of cubic In_2_O_3_ lattice, and the corresponding SAED pattern in Figure 3l recorded with an electron beam perpendicular to the surface of the In_2_O_3_ nanotower demonstrates that the In_2_O_3_ nanotower is a single crystal and the growth direction is along [200].

The growth mechanism of the In_2_O_3_ nanoneedles can be explained on the basis of the 1-D growth along the [001] crystalline direction controlled by vapor-liquid–solid (VLS) initiated due to the existence of Au catalysts [22-24]. In addition, the formation mechanism of the layered nanohooks, layered nanorods, and nanotowers is mainly led by the bottom growth of vapor-solid (VS) without a catalyst droplet [25-27]. The formation mechanism of the layered nanorods with octahedral tops is explained by the periodical 1-D growth along the [100] direction and the continuous 0-D growth along the [111] direction [14,28,29]. Beside the formation of the hook-shaped top rather than the octahedral top, the formation mechanism of the layered nanohooks is the same with the stages of the layered nanorods [14,28,29]. The formation mechanism of the nanotowers is due to a periodical 1-D growth along the [100] direction and 0-D growth along the [111] direction [14].

**Figure 4:**
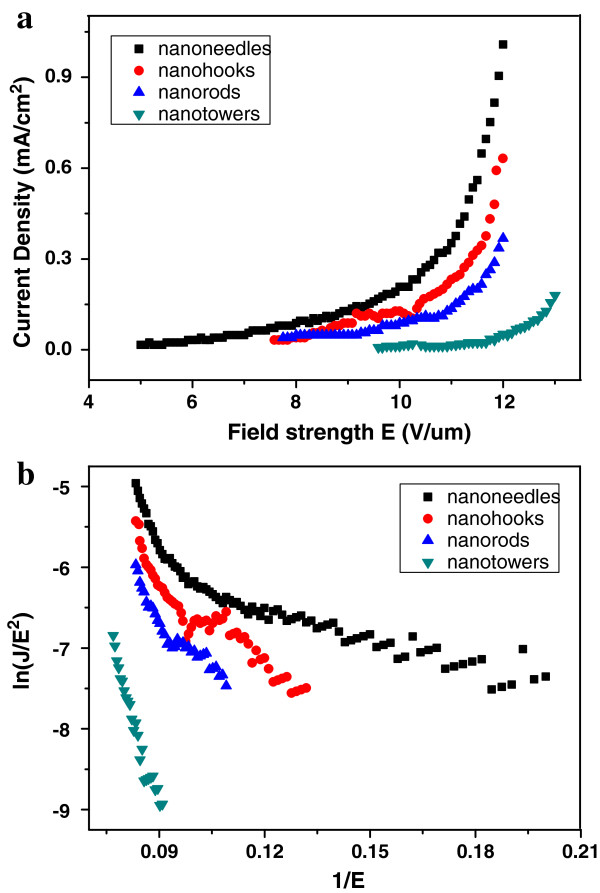
**Field emission properties of the synthesized In**_**2**_**O**_**3 **_**nanostructures. (a)** Field emission current density of the samples as a function of the electronic field. **(b)** Corresponding Fowler–Nordheim plot of the field emission current densities.

The field emission (FE) measurements of the four kinds of In_2_O_3_ nanostructures are carried out in an ultrahigh vacuum chamber at a pressure of 10^-9^ Torr at room temperature with the distance between the anode and cathode about 300 μm. Two samples with the same In_2_O_3_ nanostructures have been measured, so the number of the samples investigated is 8. The J-E properties on samples with same In_2_O_3_ nanostructures are basically uniform. From Figure 4a, we can see that the turn-on electric fields (*E*_on_) of In_2_O_3_ nanoneedles, nanohooks, nanorods, and nanotowers, which is defined as the field required to producing a current density of 10 μA/cm^2^, are 4.9, 7.5, 7.7, and 9.5 V/μm, respectively. All the applied electric fields of In_2_O_3_ nanoneedles, nanohooks, and nanorods are 12 V/μm when their current densities reach 1, 0.61, and 0.39 mA/cm^2^, respectively. So, only the In_2_O_3_ nanoneedles can obtain the threshold field (defined as the field where the current density reaches 1 mA/cm^2^) of 12 V/μm. Comparing with the turn-on electric field (defined as the field required to detect a current density of 0.1 μA/cm^2^) of 3.32 V/μm and the threshold field of 14.75 V/μm of the In_2_O_3_ awl-like structures [30], the In_2_O_3_ nanoneedles with the similar morphologies have better field emission properties. In addition, the applied electric field of the In_2_O_3_ nanotower is 13 V/μm when the current density reaches 0.16 mA/cm^2^. According to the Fowler-Nordheim (FN) theory [31], the relationship between the current density *J* and the applied field strength (*E* = V/d) can be depicted as

(1) J=Aβ2E2/Φexp-BΦ32/βE

The formula can be changed:

(2) lnJ/E2=lnAβ2/Φ-BΦ32/βE

where *A* = 1.54 × 10^-6^ A eV V^-2^, *B* = 6.83 × 10^3^ eV^-3/2^ V μm^-1^, *β* is the field enhancement factor, and Φ is the work function of an emitting material. The nonlinearity of the FN plots of the samples in Figure 4b may attribute to the space charge effects, which results from collision and ionization of residual gas molecules by the emitted electrons [32]. In addition, it has demonstrated that the different crystal facets of the emitter tip possess the different work functions [33]. According to the TEM results above, the crystal facets in the emitter tip of four kinds of In_2_O_3_ nanostructures are (001) or (100) planes, which indicates that the values of their work function are same. Assuming the work function of the In_2_O_3_ is 5.0 eV [30], *β* values of the In_2_O_3_ nanoneedles, nanohooks, nanorods, and nanotowers are estimated to be 3,695, 1,770, 1,374, and 458, respectively. Comparing with the other three kinds of In_2_O_3_ nanostructures, the In_2_O_3_ nanoneedles have the threshold field, the lowest turn-on field, and highest *β*, which demonstrates the In_2_O_3_ nanoneedles have the best field emission properties among all of the samples. The corresponding reasons can be described as follows.

It is known that the field enhancement factor *β* is a key parameter, which reflects the enhanced electron emission due to the localized electronic states by the geometrical configuration of the emitters. In theoretical case, *β* can be expressed as *h*/*r*, where *h* is the height of emitter and *r* is the average radius of the emitter tips [34]. In this paper, the In_2_O_3_ nanostructures in Figure 1 are in random alignment so that the height of emitter is difficult to measure. Based on the length of the four kinds of In_2_O_3_ nanostructures in Figure 1 being all close to 2 μm, their height of emitter can be regarded as being approximately equal. In this case, the field enhancement factor *β* is mainly depending on *1*/*r*. According to the FE mechanism, the field emission current is mainly produced from the tip of the materials so as to deduce that the field emission current is mainly produced from the tip of the nanostructures. Among the four kinds of In_2_O_3_ nanostructures in this paper, the In_2_O_3_ nanoneedles had the sharpest tip with the size of 50 nm so as to possess the highest *β* value. Therefore, the emitter tip radius and the emitter height are two factors that can affect the field emission properties of the In_2_O_3_ nanostructures.

The In_2_O_3_ nanostructures in Figure 1 are in random alignment, and the densities of the In_2_O_3_ nanostructures are all relatively high, so the screening effect between the adjacent nanostructures must be taken into account to study their field enhancement behaviors [35]. With the screening effect considered, the actual local electric field (*E*_local_) can be expressed by the Filip model [36]:

(3) $$E_{\mathrm{local}}=s\frac{V}{r}+\left(1-s\right)\frac{V}{d}$$

where *V* is the applied voltage between electrodes; *d* is the cathode–anode spacing; *r* is the emitter tip’s average radius of curvature; and *s* is a factor evaluating the degree of the screening effect, which ranges from 0 (for extremely high density emitter arrays) to 1 (for a single emitter). Apparently, the greater the *s* value is, the weaker the screening effect is. Thus, a much enhanced electric field will be obtained [35]. According to the previous reports [35,36], the relationship between *s* and the field enhancement factor *β* can be derived and formulated as

(4) $$\beta =1+s\left(\frac{d}{r}-1\right)≅1+s\frac{d}{r}$$

(5) $$s=\frac{\beta -1}{\frac{d}{r}-1}≅\left(\beta -1\right)\frac{r}{d}$$

The approximation is valid when *r* is much smaller than *d*. According to the values of *r*, *s*, and *β* in Table 1, the *s* values for the nanoneedles, nanohooks, nanorods, and nanotowers of In_2_O_3_ can be calculated as 0.307, 0.295, 0.286, and 0.229, respectively. The *s* value of the In_2_O_3_ nanoneedles is higher than the other three kinds of In_2_O_3_ nanostructures, indicating that the ability to reduce the screening effect and enhance the field emission of the In_2_O_3_ nanoneedles is better than the other three kinds of In_2_O_3_ nanostructures. Therefore, the screening effect resulting from the high density is one of the factors that can affect the field emission properties of the In_2_O_3_ nanostructures.

**Table 1:** **Field emission parameters and morphological sizes of the synthesized In **_**2**_**O**_**3 **_**nanostructures**

**Characteristics**	***r *** **(nm)**	***d *** **(μm)**	***β***	***s***
In_2_O_3_ nanoneedles	25	300	3,695	0.307
In_2_O_3_ nanohooks	50	300	1,770	0.295
In_2_O_3_ nanorods	62.5	300	1,374	0.286
In_2_O_3_ nanotowers	150	300	458	0.229

*r*, average radius of curvature at the tip; *d*, cathode–anode distance; *β*, field enhancement factor; *s*, factor evaluating the screening effect that derived from Equation 5.

In addition, different electrical properties, i.e., work function (different facet) and substrate-nanostructure electrical contact can affect the field emission properties of the In_2_O_3_ nanostructures too. According to the TEM results in Figure 3, the four kinds of In_2_O_3_ nanostructures possess the same work function due to the crystal facets in their emitter tip being (001) or (100) planes, which has been discussed above. In addition, nanostructures grown on different substrates can result in different conductivity [37]. In this paper, all of the substrates are single crystal silicon (001) substrates, so the effects of substrate-nanostructure electrical contact for the four kinds of In_2_O_3_ nanostructures are same, which may not cause the difference to their field emission properties.

From the TEM results shown in Figure 3, it is observed that the Au nanoparticles are only present at the tip of In_2_O_3_ nanoneedles. The presence of these Au nanoparticles at the tip of the nanoneedles could influence the field emission results. As the work function of Au is 5.1 eV, which is quite similar to that of In_2_O_3_. Therefore, the effect of the catalyst in the field emission properties is negligible [10].

## Conclusions

In summary, four kinds of In_2_O_3_ nanostructures, nanoneedles, layered nanohooks, layered nanorods, and nanotowers, have been grown on single silicon substrates with Au catalysts- or without any catalysts-assisted carbothermal evaporation of In_2_O_3_ and active carbon powders. The growth direction of the In_2_O_3_ nanoneedles is along the [001], and those of the other three nanostructures are along the [100]. The growth mechanism of the nanoneedles is the VLS, and those of the other three nanostructures are the VS processes. The field emission measurements demonstrated that the In_2_O_3_ nanoneedles have relatively excellent performance among the four kinds of In_2_O_3_ nanostructures mainly due to possessing the smallest emitter tip radius and the weakest screening effect.

## Competing interests

The authors declare that they have no competing interests.

## Authors’ contributions

BW carried out all the experimental processes, characterization, and mechanism research. ZQZ, HYW, LFZ conceived the study and participated in its coordination. BW drafted the manuscript. All authors read and approved the final manuscript.
